# Pain management in patients with hereditary angioedema: A case report

**DOI:** 10.1097/MD.0000000000044410

**Published:** 2026-01-23

**Authors:** Xue Li, Yanhong Ran, Chunhua Zuo, Song He, Youjia Ma, Xia Li

**Affiliations:** a Department of Gastroenterology, The Second Affiliated Hospital of Chongqing, Medical University, Chongqing, China.

**Keywords:** hereditary angioedema, MDT, multidisciplinary team, pain management

## Abstract

**Rationale:**

Hereditary angioedema (HAE) is a rare genetic disorder caused by C1 esterase inhibitor (C1-INH) deficiency or dysfunction. Presentation with isolated abdominal pain is uncommon, often leading to diagnostic delays and inadequate pain management. Effective pain control is essential to improve patient comfort and prevent complications.

**Patient concerns:**

A 21-year-old male with type II HAE presented exclusively with recurrent severe abdominal pain and experienced a transient loss of consciousness attributed to a pain-induced vagal reflex.

**Diagnoses:**

Diagnosis of type II HAE was confirmed based on clinical presentation, laboratory findings of C1-INH dysfunction, and exclusion of other causes of abdominal pain.

**Interventions:**

The patient received multidisciplinary care including symptom-based nursing, targeted pharmacological therapy with lanadelumab, psychological support, and nutritional management.

**Outcomes:**

Following lanadelumab administration, the patient’s abdominal pain improved significantly within 2 hours and completely resolved within 8 hours. Symptom relief was sustained at 3-month follow-up with no recurrence.

**Lessons:**

This case underscores the importance of early recognition of HAE presenting solely with abdominal pain and demonstrates that multidisciplinary, targeted pain management can lead to rapid and sustained symptom relief. Awareness of such atypical presentations is critical for optimizing outcomes in HAE patients.

## 1. Introduction

Hereditary angioedema (HAE) is an autosomal dominant genetic disorder caused by dysfunction or deficiency of the C1 esterase inhibitor gene (C1-INH).^[1]^ The overall prevalence of HAE is approximately 1 in 50,000 to 1 in 100,000.^[1,2]^ HAE is primarily classified into 3 subtypes: type I, type II, and type III, with type II HAE accounting for approximately 15% of all HAE cases.^[3]^ Notably, in China, type II HAE is even rarer, with a prevalence of only 5% of all HAE cases.^[4]^

Studies have shown that the clinical manifestations of HAE include recurrent swelling of various body parts, such as the face, extremities, gastrointestinal tract, and respiratory tract.^[5]^ Among HAE patients, approximately 60% to 70% experience abdominal symptoms, while only 30% present with symptoms confined solely to the abdomen.^[5]^ During abdominal attacks, swelling of the intestinal wall can lead to excruciating pain, causing significant distress, weakness, and a profound impact on patients’ daily lives and work.^[5]^ The ultimate treatment goal for HAE is to control symptom occurrence, reduce the frequency and severity of attacks, and enable patients to lead a normal life.^[6]^ For HAE patients whose symptoms are limited to abdominal pain, effective pain management becomes particularly crucial.

In this report, we present a case of an HAE patient with symptoms confined exclusively to abdominal pain and discuss the strategies employed for pain management. This case provides valuable insights and practical reference for improving the management of abdominal pain in similar HAE cases in the future.

## 2. Case report

The patient, a 21-year-old male, presented with severe cramping pain in the epigastric and mid-upper abdominal regions 3 days prior to admission, without any apparent triggers. Despite receiving antiinfective and antispasmodic treatment at a local hospital, his symptoms showed no significant improvement. He subsequently visited the emergency department of our hospital and was admitted to our department with a primary complaint of “abdominal pain.” The patient had a history of depression with multiple suicide attempts. On admission, physical examination revealed generalized abdominal tenderness, rebound tenderness, and muscular guarding, accompanied by back pain, vomiting (with gastric contents), and cessation of defecation and flatus. Abdominal computed tomography (CT) demonstrated diffuse thickening and edema of the small intestinal wall, along with abdominal and pelvic effusion. A preliminary diagnosis of abdominal pain of uncertain etiology was made, with differential diagnoses including inflammatory bowel disease, immune-related disease, or vascular disease.

The patient experienced significant abdominal pain after admission, with a pain score of 7 out of 10 using the Numerical Rating Scale (NRS), indicating severe pain. On the third day of admission at 17:35, the patient experienced a sudden loss of consciousness lasting approximately 30 seconds, followed by spontaneous recovery. This episode was accompanied by increased respiratory and heart rates and was suspected to be caused by a vagal reflex triggered by severe pain. A multidisciplinary consultation raised a high suspicion of HAE. Further laboratory testing revealed the following results: C1 esterase inhibitor function at 45.38%, C1 esterase inhibitor concentration at 104.61 µg/mL, and complement C4 at 23.63 µg/mL. Combined with the patient’s clinical symptoms and CT findings of diffuse thickening and edema of the small intestinal wall, a definitive diagnosis of type II hereditary angioedema (HAE-II) was made. A multidisciplinary team, including medical staff, nurses, nutritionists, pharmacists, and psychological therapists, collaborated to provide the patient with comprehensive management. Interventions included gastrointestinal care, respiratory training, pain position management, pharmacological treatment, nutritional support, and narrative psychological care. By the fifth day of hospitalization, the patient’s abdominal pain had significantly improved.

Prior to lanadelumab infusion, his NRS pain score fluctuated between 6 to 8/10 despite sequential use of tramadol and pethidine. The analgesic effect lasted for approximately 1 hour, after which the pain gradually worsened. Following intravenous administration of lanadelumab, the NRS score decreased from 7/10 to 2/10 within 120 minutes and achieved complete resolution (0/10) within 8 hours. No further episodes of severe pain occurred during hospitalization. Due to financial constraints, the patient was transferred to a county-level hospital on the sixth day for continued treatment, which lasted 1 week. On the second day at the county hospital, he transitioned from intravenous nutritional support to oral feeding and was able to walk independently without symptom exacerbation. At discharge, he reported no abdominal pain or vomiting and was tolerating a normal diet. At the 3-month follow-up, the patient reported only 2 episodes of mild abdominal discomfort (NRS 2/10), both of which resolved spontaneously without medication. No hospitalizations, emergency department visits, or drug-related adverse reactions were recorded during the follow-up period.

## 3. Discussion

Through a review of the literature, we found that although there has been increasing scholarly focus on the treatment of HAE in recent years,^[7–9]^ articles specifically addressing pain management in HAE patients remain relatively scarce. This may be attributed to the fact that fewer than one-third of diagnosed HAE patients present with symptoms confined solely to abdominal pain.^[5]^ Moreover, compared to abdominal pain, laryngeal edema poses a more immediate life-threatening risk to patients, likely garnering greater attention from researchers.^[10]^ Studies indicate that while abdominal pain in HAE is typically self-limiting, the associated symptoms can be extremely severe, significantly impairing patients’ quality of life.^[5]^ For instance, in the present case, the patient experienced transient loss of consciousness due to severe abdominal pain. Therefore, abdominal pain in HAE patients also warrants close attention from healthcare teams.

## 4. Timely and accurate diagnosis as the foundation for managing abdominal pain

Timely and accurate diagnosis is critical for managing abdominal pain in HAE patients. Studies have shown that the rarity of HAE increases diagnostic challenges, particularly in cases where symptoms are confined solely to abdominal pain. Such cases can easily be mistaken for other acute abdominal conditions, leading to diagnostic delays or even unnecessary surgical interventions.^[11]^ National surveys conducted in Denmark and Spain revealed that the average diagnostic delay for HAE is approximately 13 to 16 years.^[12,13]^ Additionally, studies in Japan and the United States reported that only 12% of patients are diagnosed with HAE during their first visit to a healthcare facility. On average, patients must consult 4.4 to 4.6 different specialists before receiving a definitive diagnosis.^[14,15]^ In this case, given the patient’s acute onset, rapid disease progression, atypical presentation, and unclear etiology of abdominal pain, our team promptly initiated a hospital-wide consultation. Through the collaborative efforts of a multidisciplinary team, differential diagnoses such as intestinal tuberculosis, allergic diseases, systemic lupus erythematosus, and inflammatory bowel disease were systematically excluded. Ultimately, the patient was successfully diagnosed with type II HAE during their first episode of symptoms.

This timely and accurate diagnosis laid a crucial foundation for the effective management of the patient’s abdominal pain.

## 5. Understanding the mechanism of abdominal pain in HAE: a key to symptom management

Studies have shown that type II hereditary angioedema (HAE-II) is characterized by the production of nonfunctional C1 esterase inhibitor protein. Dysfunctional C1 inhibitor leads to uncontrolled complement activation, which promotes vascular permeability and edema.^[16]^ Furthermore, the deficiency in C1 inhibitor function results in excessive activation of the complement system and overactivation of kallikrein, leading to an overproduction of bradykinin. Bradykinin acts on B2 receptors, increasing endothelial permeability and causing edema in the gastrointestinal mucosa and serosa. This mechanism is the most direct cause of abdominal pain in HAE-II patients.^[17]^ In this case, the patient’s CT scans demonstrated diffuse thickening and edema of the small intestinal wall, accompanied by abdominal and pelvic effusion. Gastrointestinal mucosal edema and effusion stimulation were identified as the fundamental causes of the severe abdominal pain experienced by the patient.

## 6. Multidisciplinary team collaboration in pain management

Compared to traditional single-discipline medical approaches, multidisciplinary team (MDT) collaboration integrates expertise from various fields, enabling comprehensive assessment of a patient’s condition and providing precise, individualized treatment and care plans.^[18]^ MDT-based disease management strategies have been widely implemented in many countries, yielding positive outcomes in both diagnosis and treatment. For instance, a study in the United States demonstrated the critical role of MDT collaboration in managing patients with hepatocellular carcinoma. By combining multidisciplinary expertise, the approach improved diagnostic accuracy and patient treatment outcomes.^[19]^ Similarly, a study from China highlighted that MDT collaboration improved the quality of care for patients with brain metastases from breast cancer, significantly enhancing survival rates.^[20]^ In the present case, the patient presented with unexplained abdominal pain. A multidisciplinary team comprising physicians, nurses, pharmacists, nutritionists, and psychotherapists collaborated to manage the patient’s pain. This comprehensive, team-based approach ultimately led to significant relief of the patient’s abdominal pain.

In the management of acute abdominal pain, withholding oral intake (NPO) helps to reduce gastrointestinal burden, alleviate vomiting symptoms, and minimize the risk of aspiration.^[21]^ In this case, the patient maintained NPO status from the time of admission. To ensure the patient’s full compliance with the NPO decision, our nutritionist developed an individualized nutritional infusion plan. Given the patient’s characteristics – 21-year-old male, height 174 cm, weight 54 kg, with a BMI of 17.84 kg/m² – we calculated his daily caloric requirements to be 1930 kcal, with 76 g of protein (the detailed parenteral nutrition formula is provided in Supplement 1, Supplemental Digital Content, https://links.lww.com/MD/R232). Using an infusion pump, we controlled the infusion rate to complete the nutritional support within 12 hours. During the NPO period, the patient adhered well to the plan, reporting no hunger discomfort and no incidents of hypoglycemia. This nutritional support laid the foundation for effective gastrointestinal management of the patient.

Another study has shown that nasogastric tube decompression can help remove excess gas and fluid from the gastrointestinal tract, thereby reducing intestinal pressure and alleviating abdominal pain.^[22]^ In the present case, the patient presented with vomiting and small bowel edema. Upon admission, we instructed the patient to remain NPO and immediately placed a nasogastric tube for gastrointestinal decompression. At the same time, we attempted to use diaphragmatic breathing to modulate autonomic nervous function, aiming to influence the gastrointestinal system and help the patient alleviate pressure and improve pain.^[23]^ However, despite these efforts, neither of these interventions demonstrated significant therapeutic effects in this patient.

**Figure 1.**
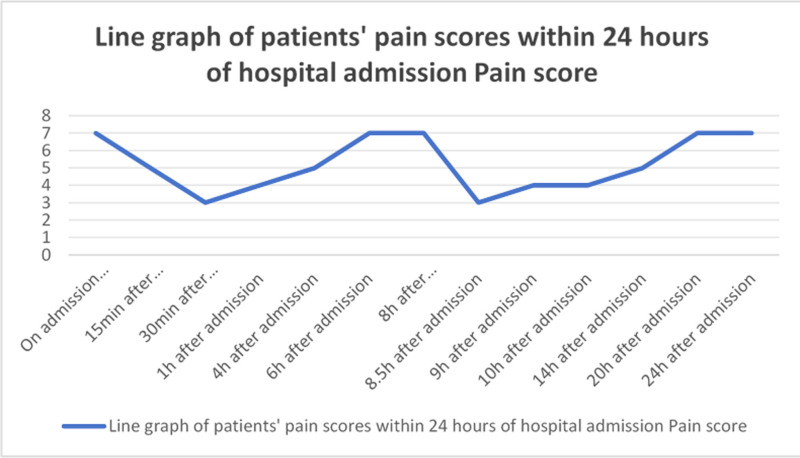
Line graph of the patient’s pain score 24 h prior to hospital admission.

**Figure 2.**
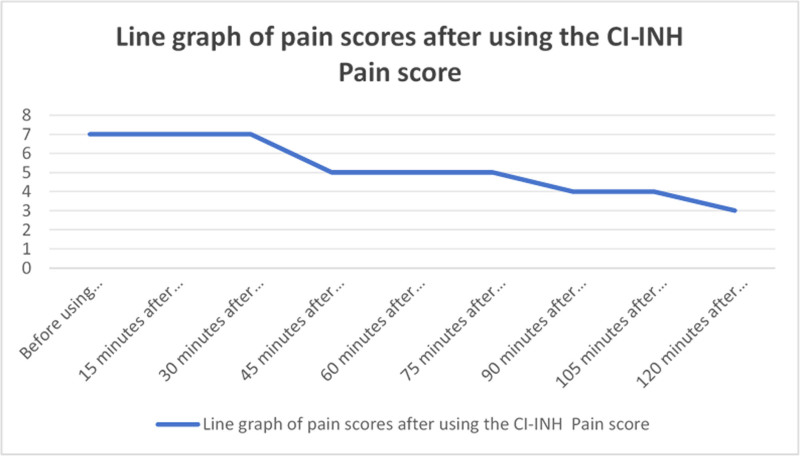
Line graph of pain scores after using the CI-INH. C1-INH = C1 esterase inhibitor.

To further alleviate the patient’s abdominal pain symptoms, within 48 hours of admission, a decision was made by the medical team, including physicians, nurses, and pharmacists, to sequentially administer tramadol and dextromethorphan for pain relief. However, after administration, we observed that the analgesic effects lasted only for 1 hour, after which the pain gradually worsened, with the patient’s pain scores fluctuating in a wave-like pattern (see Fig. 1). On the third day of admission, the patient experienced a transient loss of consciousness due to pain-induced vagal stimulation. Following a discussion within the multidisciplinary team (MDT) consisting of physicians, nurses, pharmacists, and psychological counselors, HAE type II was highly suspected. The 2021 WAO/EAACI guidelines also recommend that when HAE is clinically suspected, specific treatment should be initiated immediately, without waiting for laboratory results.^[24]^ To prevent further deterioration of the patient’s condition, our team decided to administer lanadelumab. Within 2 hours of administration, we conducted repeated pain assessments every 15 minutes. We observed that the patient’s abdominal pain was significantly controlled by the 120-minute mark, and it was completely alleviated within 8 hours post-infusion (see Fig. 2).

Research indicates that patients with HAE often experience significant anxiety and depression,^[25]^ and increased anxiety can exacerbate pain perception and reduce pain tolerance. Therefore, effective psychological interventions are crucial in managing pain in HAE patients.^[26]^ Narrative nursing interventions have been shown to significantly improve patients’ anxiety and depression, as well as reduce their subjective pain experience.^[27]^ In this case, our narrative nursing team, consisting of narrative care nurses and psychologists, closely collaborated to provide interventions. On the first, third, and fourth days of the patient’s hospitalization, narrative nursing interviews were conducted with both the patient and the primary care nurses. By listening to and understanding the patient’s life story, the nursing staff were able to better empathize with the patient’s suffering and needs, which allowed for the identification of key care issues. This approach led to the design of more tailored care strategies to meet the patient’s individual needs. By sharing their stories, the patient expressed inner needs and expectations, which enhanced the patient’s autonomy and self-decision-making during the treatment process, thereby improving treatment adherence. Additionally, the psychological relief gained from the narrative intervention increased the patient’s pain tolerance, thus supporting effective symptom management.

## 7. Limitations

This case report has limitations due to its single-patient design, which may restrict its generalizability. In addition, the patient was discharged from treatment due to financial issues, limiting our ability to provide comprehensive diagnosis, treatment, and care. Furthermore, follow-up was limited to 3 months, restricting our ability to assess the long-term efficacy of treatment.

## 8. Conclusion

The pain management experience in this HAE patient demonstrates that multidisciplinary collaboration and effective pain management significantly improve pain symptoms and contribute to the overall recovery of the patient. This experience highlights that, although abdominal pain may be a self-limiting manifestation of HAE, timely and accurate diagnosis, appropriate symptom-based nursing interventions, psychological support, and specific treatment are crucial to the patient’s comfort and rapid recovery. Future clinical practice should focus on further optimizing abdominal pain management strategies for hereditary angioedema patients, ensuring that these patients receive comprehensive treatment and care, ultimately aiming to improve their prognosis and quality of life.

## Author contributions

**Conceptualization:** Xia Li.

**Formal analysis:** Yanhong Ran, Chunhua Zuo.

**Investigation:** Song He.

**Methodology:** Yanhong Ran, Chunhua Zuo.

**Resources:** Youjia Ma, Xia Li.

**Visualization:** Youjia Ma.

**Writing – original draft:** Xue Li, Xia Li.

**Writing – review & editing:** Xue Li, Xia Li.

## Supplementary Material



## References

[1] Wilkerson RG Moellman JJ. Hereditary angioedema. Immunol Allergy Clin North Am. 2023;43:533–52. 37394258 10.1016/j.iac.2022.10.012

[2] Nasr IH Manson AL Wahshi HAA Longhurst HJ. Optimizing hereditary angioedema management through tailored treatment approaches. Expert Rev Clin Immunol. 2016;12:19–31. 26496459 10.1586/1744666X.2016.1100963

[3] Patel G Pongracic JA. Hereditary and acquired angioedema. Allergy Asthma Proc. 2019;40:441–5. 31690390 10.2500/aap.2019.40.4267

[4] Cao Y Liu S Zhi Y. Recurrent and acute abdominal pain as the main clinical manifestation in patients with hereditary angioedema. Allergy Asthma Proc. 2021;42:131–5. 33685557 10.2500/aap.2021.42.210001 PMC8133019

[5] Cicardi M Aberer W Banerji A; HAWK under the patronage of EAACI (European Academy of Allergy and Clinical Immunology). Classification, diagnosis, and approach to treatment for angioedema: consensus report from the Hereditary Angioedema International Working Group. Allergy. 2014;69:602–16. 24673465 10.1111/all.12380

[6] Maurer M Magerl M Betschel S. The international WAO/EAACI guideline for the management of hereditary angioedema-The 2021 revision and update. Allergy. 2022;77:1961–90. 35006617 10.1111/all.15214

[7] Gower RG Wilber M. Considerations for transition from subcutaneous to oral prophylaxis in the treatment of hereditary angioedema. Allergy Asthma Clin Immunol. 2021;17:100. 34627358 10.1186/s13223-021-00603-9 PMC8501591

[8] Bernardino AG Ferreira MB Costa C Caiado J Pedro E Santos AS. Experience of lanadelumab administration in hereditary angioedema: a case series of 4 patients in Portugal. Asia Pacific allergy. 2023;13:91–4. 37388816 10.5415/apallergy.0000000000000102 PMC10287104

[9] Staikuniene-Kozonis J Staikunaite J Gasiuniene E Sematonyte J. Case report: early presentation of hereditary angioedema symptoms in a 2-year-old boy. Front Pediatr. 2024;12:1408110. 38978843 10.3389/fped.2024.1408110 PMC11228810

[10] Andrejević S Korošec P Šilar M. Hereditary angioedema due to C1 inhibitor deficiency in serbia: two novel mutations and evidence of genotype-phenotype association. PLoS One. 2015;10:e0142174. 26535898 10.1371/journal.pone.0142174 PMC4633032

[11] Frank MM. Hereditary angioedema: the clinical syndrome and its management in the United States. Immunol Allergy Clin North Am. 2006;26:653–68. 17085283 10.1016/j.iac.2006.09.005

[12] Bygum A. Hereditary angio-oedema in Denmark: a nationwide survey. Br J Dermatol. 2009;161:1153–8. 19709101 10.1111/j.1365-2133.2009.09366.x

[13] Roche O Blanch A Caballero T Sastre N Callejo D López-Trascasa M. Hereditary angioedema due to C1 inhibitor deficiency: patient registry and approach to the prevalence in Spain. Ann Allergy Asthma Immunol. 2005;94:498–503. 15875532 10.1016/S1081-1206(10)61121-0

[14] Lunn ML Santos CB Craig TJ. Is there a need for clinical guidelines in the United States for the diagnosis of hereditary angioedema and the screening of family members of affected patients? Ann Allergy Asthma Immunol. 2010;104:211–4. 20377110 10.1016/j.anai.2009.12.004

[15] Iwamoto K Yamamoto B Ohsawa I. The diagnosis and treatment of hereditary angioedema patients in Japan: a patient reported outcome survey. Allergology Int. 2021;70:235–43. 10.1016/j.alit.2020.09.008 33168485

[16] Sinnathamby ES Issa PP Roberts L. Hereditary angioedema: diagnosis, clinical implications, and pathophysiology. Adv Ther. 2023;40:814–27. 36609679 10.1007/s12325-022-02401-0 PMC9988798

[17] Zuraw BL Christiansen SC. HAE pathophysiology and underlying mechanisms. Clin Rev Allergy Immunol. 2016;51:216–29. 27459852 10.1007/s12016-016-8561-8

[18] Taberna M Gil Moncayo F Jané-Salas E. The multidisciplinary team (MDT) approach and quality of care. Front Oncol. 2020;10:85. 32266126 10.3389/fonc.2020.00085 PMC7100151

[19] Siddique O Yoo ER Perumpail RB. The importance of a multidisciplinary approach to hepatocellular carcinoma. J Multidiscip Healthc. 2017;10:95–100. 28360525 10.2147/JMDH.S128629 PMC5365324

[20] Xu F Ou D Qi W. Impact of multidisciplinary team on the pattern of care for brain metastasis from breast cancer. Front Oncol. 2023;13:1160802. 37664027 10.3389/fonc.2023.1160802 PMC10471195

[21] Soni P Kumar V Alliu S Shetty V. Hereditary angioedema (HAE): a cause for recurrent abdominal pain. BMJ Case Rep. 2016;2016:bcr2016217196. 10.1136/bcr-2016-217196 PMC5129146 27873761

[22] Fu JH Zhao N Liu B. [Advances in clinical application of obstruction catheter in prevention and treatment of intestinal obstruction]. Zhonghua wei chang wai ke za zhi. 2021;24:931–5. 34674470 10.3760/cma.j.cn.441530-20200305-00120

[23] Hamasaki H. Effects of diaphragmatic breathing on health: a narrative review. Medicines (Basel). 2020;7:65. 33076360 10.3390/medicines7100065 PMC7602530

[24] Maurer M Aygören-Pürsün E Banerji A. Consensus on treatment goals in hereditary angioedema: a global Delphi initiative. J Allergy Clin Immunol. 2021;148:1526–32. 34048855 10.1016/j.jaci.2021.05.016

[25] Fouche AS Saunders EF Craig T. Depression and anxiety in patients with hereditary angioedema. Ann Allergy Asthma Immunol. 2014;112:371–5. 24428960 10.1016/j.anai.2013.05.028 PMC4211935

[26] Michaelides A Zis P. Depression, anxiety and acute pain: links and management challenges. Postgrad Med. 2019;131:438–44. 31482756 10.1080/00325481.2019.1663705

[27] Wen B Liu Y Min XX Wang AQ. Nursing effect of narrative nursing intervention on postoperative patients with severe lung cancer. World J Clin Cases. 2024;12:76–85. 38292623 10.12998/wjcc.v12.i1.76 PMC10824191

