# Supplementary material for: Pain management in patients with hereditary angioedema: A case report
Source: Medicine (Baltimore). 2026 Jan 23;105(4):e44410. doi: 10.1097/MD.0000000000044410 (PMC12851712; doi:10.1097/MD.0000000000044410)
Supplement: Supplementary file 1 [file medi-105-e44410-s001.pdf]

Supplement 1: Parenteral Nutrition Ratios

Name	Dosages
50% glucose	350ml
10% glucose	100ml
Lovan Life Amino Acids	750ml
Multiple fatty milks	350ml
water-soluble vitamins	1 stick
fat-soluble vitamins	1 stick
Andamex	1 stick
15%KCL	20ml
10%Nacl	40ml
